# Laparoscopic Management of Cornual Ectopic Pregnancy: A Narrative Review

**DOI:** 10.7759/cureus.104080

**Published:** 2026-02-22

**Authors:** Haydee S Sosa-Castillo, Luz M Bravo-Rodriguez

**Affiliations:** 1 Gynecological Endoscopic Surgery, Mexican Faculty of Medicine, La Salle University, Mexico City, MEX; 2 Gynecological Endoscopic Surgery Service, Lic. Adolfo López Mateos Regional Hospital, Institute for Social Security and Services for State Workers (ISSSTE), Mexico City, MEX

**Keywords:** cornual ectopic pregnancy, cornuostomy, hemostatic techniques, interstitial pregnancy, laparoscopic cornual resection, laparoscopic management, laparoscopy, purse-string suture, vasopressin injection, wedge resection

## Abstract

Cornual ectopic pregnancy is a rare obstetric condition characterised by high diagnostic complexity and a significant risk of serious haemorrhagic complications. The specific vascularisation of the uterine horn increases the likelihood of uterine rupture, typically occurring in later stages of pregnancy and leading to higher maternal morbidity. In this context, advances in minimally invasive surgery have established the laparoscopic approach as a treatment option for selected patients. This narrative review aims to synthesise the available evidence regarding the laparoscopic management of cornual ectopic pregnancy by analysing the anatomical and pathophysiological basis, the main diagnostic challenges, the therapeutic alternatives, and the laparoscopic surgical techniques currently in use. The review also examines reported clinical outcomes, the profile of complications, and considerations related to fertility preservation. Based mainly on evidence derived from case reports, small case series, and retrospective observational studies, laparoscopic treatment performed by experienced teams appears to be associated with favourable clinical outcomes and acceptable safety profiles in selected patients; however, the heterogeneity and limited level of evidence underscore the need for further comparative and prospective studies.

## Introduction and background

Definition

A cornual ectopic pregnancy is a rare type of ectopic pregnancy involving implantation of the gestational sac in the cornual region of the uterus. This region is anatomically related to the interstitial segment of the fallopian tube that traverses the uterine myometrium [1,2]. This location is characterised by proximity to the myometrial tissue and rich vascularisation, which allows the gestational sac to expand before rupture, typically between weeks 7 and 16 of gestation [1,3].

From an anatomical perspective, the interstitial segment of the fallopian tube measures approximately 1-2 cm and is surrounded by myometrium. This anatomical feature complicates early diagnosis and explains the later clinical presentation compared with other tubal ectopic pregnancies [2,4]. Due to this complexity, the terms 'cornual' and 'interstitial' pregnancy have often been used interchangeably. However, several authors recommend reserving the term 'cornual pregnancy' for pregnancies implanted in a uterine horn associated with Müllerian anomalies, such as a rudimentary horn of a unicornuate uterus [3,5].

For the purposes of this review, the term 'cornual/interstitial ectopic pregnancy' will be used consistently to reflect common clinical usage while acknowledging existing terminological debate. Regardless of terminology, this condition is associated with a high risk of uterine rupture and severe haemorrhage, with maternal mortality rates higher than those observed in other ectopic locations [1,4,6].

Epidemiology

Ectopic pregnancy accounts for approximately 1% to 2% of all pregnancies and remains an important cause of first-trimester maternal morbidity and mortality [7,8]. More than 90% to 95% occur in the fallopian tube, whereas non-tubal locations represent a smaller but clinically more complex group [8,9]. Although these non-tubal locations constitute a minority of cases, they disproportionately contribute to severe haemorrhagic complications and maternal mortality, which underscores the need for early recognition and specialised management.

Cornual/interstitial ectopic pregnancy accounts for approximately 2% to 4% of ectopic pregnancies, with an estimated incidence of one in 2,500-5,000 live births [1,10,11]. Despite its rarity, it carries a disproportionately high risk of uterine rupture and massive haemorrhage, with reported maternal mortality rates ranging from 2% to 2.5% [4,11].

An increase in incidence has been described in recent decades, mainly related to assisted reproductive technologies, previous tubal surgery, prior salpingectomy, and underlying tubal pathology [10,11,12]. In this context, heterotopic and atypical implantations are more frequently encountered [12].

Beyond epidemiological figures, the clinical impact of cornual/interstitial pregnancy varies significantly across healthcare systems. In centres lacking immediate access to minimally invasive surgery, interventional radiology, or blood bank support, delayed diagnosis may translate into higher morbidity and mortality. Therefore, understanding this condition requires not only anatomical knowledge but also awareness of resource-dependent management realities, which strongly influence therapeutic options and outcomes.

The tendency for delayed diagnosis related to myometrial distensibility further increases clinical risk and maintains this entity as a major diagnostic challenge despite advances in transvaginal ultrasound and serial beta-human chorionic gonadotropin (β-hCG) monitoring [8,13].

Clinical risk

Cornual/interstitial ectopic pregnancy carries a particularly high clinical risk profile compared with other ectopic locations due to both anatomical and pathophysiological factors. Implantation within the interstitial segment is surrounded by thick myometrium and receives abundant uterine and ovarian vascular supply, allowing progressive distension before rupture [1,10].

Consequently, uterine rupture often occurs later in gestation (seven to 16 weeks) and may present abruptly with massive haemorrhage and rapid haemodynamic deterioration [1,11]. This explains why maternal mortality rates exceed those of ampullary ectopic pregnancy [4,11].

Clinical manifestations are frequently non-specific, including mild pelvic pain and light vaginal bleeding [7,8], contributing to diagnostic delays. Even initially stable patients may rapidly deteriorate with haemoperitoneum and hypovolaemic shock [6]. Risk is further increased among patients with previous tubal surgery, assisted reproduction, or altered pelvic anatomy [10,11].

To improve translational utility, risk assessment should consider not only diagnosis but also immediate clinical status and treatment context. Practical risk stratification assists clinicians in deciding between conservative treatment, planned minimally invasive surgery, or emergency laparotomy (Table 1). 

**Table 1:** Practical clinical risk stratification in cornual/interstitial ectopic pregnancy β-hCG: beta-human chorionic gonadotropin

Clinical status	Typical features	Management considerations
Low immediate risk	Hemodynamically stable, small gestation, no rupture	Conservative or minimally invasive options in experienced centres
Intermediate risk	Stable but larger gestation, high β-hCG, uncertain imaging	Early referral, specialist imaging, and planned surgical management
High risk	Hemodynamic instability, suspected rupture, haemoperitoneum	Emergency surgery (often laparotomy), rapid haemorrhage control

A laparoscopic approach to cornual ectopic pregnancy

Surgical management has evolved substantially over recent decades, moving from traditional laparotomy toward minimally invasive approaches. Historically, concern regarding uncontrolled bleeding often required radical procedures, including cornuectomy or hysterectomy, particularly in emergency settings [10,14].

Evidence supporting laparoscopic management mainly derives from retrospective case series and technical reports [15,16,17], while comparative studies remain limited, and high-level evidence is scarce. Nevertheless, these reports suggest that laparoscopy can provide effective haemostasis, reduced postoperative morbidity, and faster recovery in appropriately selected haemodynamically stable patients [15,16]. This approach is particularly relevant for women wishing to preserve fertility [18].

Different laparoscopic techniques have been described, including cornuostomy and wedge resection. Their selection depends on gestational size, rupture status, surgical expertise, and patient characteristics [14,18]. Haemostatic strategies such as intramyometrial vasopressin injection and purse-string sutures are commonly described in technical reports and case series [10,19].

Reported surgical success rates are largely derived from small case series and retrospective reviews rather than randomised comparisons [15,16,18]. Although promising outcomes have been published, laparoscopy remains highly dependent on resource availability and surgical experience. In many healthcare settings globally, laparotomy continues to represent a critical life-saving option, particularly when advanced minimally invasive resources or blood bank support are limited.

Compared with medical treatment, laparoscopy offers immediate resolution and direct haemostatic control, particularly in cases with elevated β-hCG levels or embryonic cardiac activity [10,20]. However, therapeutic decisions must be individualised according to clinical stability and institutional capability.

**Figure 1:**
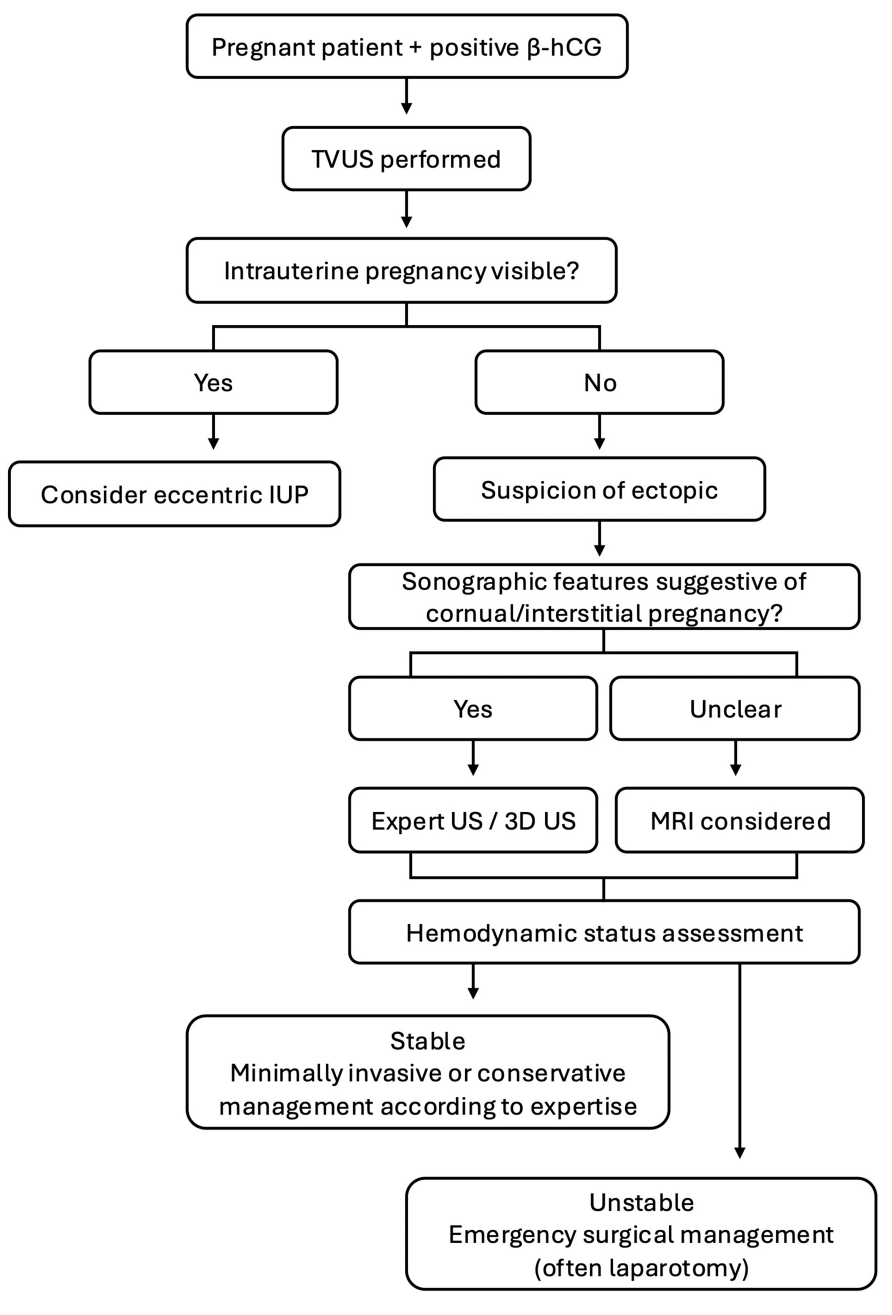
Proposed diagnostic and initial management pathway for suspected cornual/interstitial ectopic pregnancy Overall, the available evidence suggests that laparoscopy represents an important therapeutic option when performed in specialised settings, but current data largely originate from lower-level evidence studies. Therefore, clinical decisions should balance patient status, surgeon expertise, and healthcare system resources. β-hCG: beta-human chorionic gonadotropin; TVUS: transvaginal ultrasound; IUP: intrauterine pregnancy; US: ultrasound This image has been created by the authors based on the literature discussed in this review.

To contextualise diagnostic and early management pathways across different clinical settings, a proposed practical algorithm is shown in Figure 1. 

## Review

Diagnostic considerations 

The diagnosis of cornual ectopic pregnancy poses a significant clinical challenge due to its unique anatomical location and the similarity of its symptoms to those of other types of ectopic pregnancy and variants of abnormal intrauterine implantation. Although transvaginal ultrasound is the primary diagnostic tool, accurately identifying the cornual or interstitial location can be difficult, particularly in the early stages of gestation and in patients with intact uterine anatomy [8, 10, 21].

The ultrasound criteria most frequently described include visualisation of an eccentric gestational sac separated from the endometrial cavity and located in the superolateral region of the uterus, surrounded by a thin and incomplete layer of myometrium. Additionally, the interstitial line sign has been described as a guiding finding for interstitial or cornual pregnancy, although its sensitivity varies depending on gestational age and operator experience [8, 22, 23]. From an evaluative perspective, transvaginal ultrasound remains the first-line modality because of its wide availability and rapid bedside applicability; however, diagnostic accuracy is strongly dependent on operator expertise and anatomical conditions, which may reduce sensitivity in early gestation or atypical implantation sites. Three-dimensional ultrasound may improve spatial assessment of the gestational sac in relation to the endometrial cavity and surrounding myometrium and can increase diagnostic confidence in equivocal cases, although robust comparative accuracy data remain limited [23]. Magnetic resonance imaging is generally reserved for selected situations in which ultrasound findings are inconclusive or when distinguishing cornual/interstitial pregnancy from angular or eccentric intrauterine pregnancy would significantly modify management decisions, particularly regarding conservative monitoring versus surgical intervention [11, 23, 24, 25]. Overall, although these imaging modalities are complementary, high-quality comparative studies evaluating sensitivity and specificity across techniques remain scarce, and diagnostic interpretation continues to rely heavily on expert imaging assessment integrated with clinical context [8, 22, 23].

However, several authors agree that these ultrasound criteria do not always permit conclusive differentiation, which is why a significant proportion of cases are definitively diagnosed during surgery. This diagnostic limitation is exacerbated by the ongoing confusion surrounding the terminology of cornual, interstitial, and angular pregnancies, as documented in numerous radiological and obstetric publications. This confusion can lead to errors in diagnosis with significant therapeutic consequences [10, 24].

The use of complementary imaging modalities has become more important in recent years. Three-dimensional ultrasound provides a clearer view of the gestational sac in relation to the endometrial cavity and the surrounding myometrial thickness. Magnetic resonance imaging has also been suggested as a useful tool in certain cases, especially when two-dimensional ultrasound fails to determine the exact location of the implantation site [11, 23, 24, 25].

A delayed diagnosis can have critical clinical implications, given that a cornual ectopic pregnancy can progress silently for a longer period than an ampullary tubal ectopic pregnancy due to the distensibility of the interstitial myometrium. This characteristic favours a late clinical presentation and is associated with a high risk of catastrophic uterine rupture and massive haemorrhage [7, 13, 26, 27].

Several recent studies emphasise the importance of integrating clinical findings, serum β-hCG levels, and systematic ultrasound evaluation for optimising early diagnosis. However, it has been pointed out that β-hCG values above the discriminatory range alone do not allow a reliable distinction to be made between an eccentric intrauterine pregnancy and a true cornual pregnancy. This reinforces the need for expert imaging evaluation [21, 26, 27].

Consequently, several authors emphasise the importance of maintaining a high level of clinical suspicion, even in patients who are haemodynamically stable and exhibit non-specific symptoms, particularly if they have risk factors such as undergoing assisted reproductive techniques, having a history of tubal surgery, or having had a previous ectopic pregnancy. In this context, an accurate and timely diagnosis is crucial in reducing the morbidity and mortality associated with this condition, as well as in guiding subsequent therapeutic strategies [7, 21, 24].

Therapeutic options

The management of cornual ectopic pregnancy can include medical, surgical, or combined approaches. The choice of approach must be carefully tailored to the individual patient and based on various clinical factors, including their haemodynamic status, gestational age, serum β-hCG levels, the presence of embryonic cardiac activity, the size and exact location of the gestational sac, and their future reproductive desires [10, 20, 28]. In practical terms, management follows a stepwise clinical hierarchy in which haemodynamic stability constitutes the primary decision point. Haemodynamically unstable patients or those with suspected rupture generally require immediate surgical intervention, whereas stable patients may be considered for conservative or minimally invasive strategies depending on imaging findings, available expertise, and institutional resources.

Medical treatment with methotrexate is widely recognised as a conservative alternative in selected cases, particularly for patients who are haemodynamically stable, have received an early diagnosis, have small gestational sacs, and have relatively low β-hCG levels. However, the available evidence suggests variable success rates and an appreciable proportion of therapeutic failures, even in seemingly favourable contexts. Several authors have documented the persistent risk of late uterine rupture following medical treatment, even when an initial decrease in β-hCG levels is observed. This limits the universal application of this treatment in this specific location [8, 20, 29, 30].

In addition, combined therapeutic regimens have been proposed, including the systemic or local administration of methotrexate. In some cases, this is associated with mifepristone or minimally invasive, image-guided procedures. While these strategies have shown encouraging results in carefully selected patients, mainly in case reports and small series, the lack of robust comparative studies prevents definitive recommendations from being established [30, 31, 32].

Traditionally, surgical management of cornual ectopic pregnancy involved laparotomy, particularly in cases of uterine rupture, haemodynamic instability, or massive haemorrhage. While this approach is effective in immediately controlling bleeding, it is associated with higher morbidity, prolonged hospital stays, and a potentially negative impact on future fertility [14, 33]. Importantly, laparotomy remains a critical option in healthcare systems where minimally invasive surgery or specialised resources are not immediately available, highlighting the need to individualise therapeutic decisions according to local infrastructure and surgical capability.

Advances in minimally invasive surgery and improvements in laparoscopic techniques have established laparoscopy as a preferred alternative for selected patients, even in complex situations. Evidence supporting laparoscopic management mainly derives from retrospective case series and technical reports [14,15,34], while comparative studies remain limited [33], and high-level evidence is scarce. Reported advantages include less blood loss, reduced postoperative pain, faster recovery, and preservation of uterine anatomy, particularly when procedures are performed by experienced minimally invasive surgeons [14,15,33,34].

Laparoscopic surgical options include cornuostomy and wedge resection of the cornu. In selected cases, procedures can be combined with advanced haemostatic techniques or uterine artery embolisation. The choice of technique depends on the size of the pregnancy, the depth of myometrial invasion, the presence of active bleeding, and the experience of the surgical team. Recent reports suggest that laparoscopy can be used in certain cases of ruptured cornual pregnancy in highly specialised centres with appropriate expertise and resources [32, 34]. 

Table 2 summarises the main therapeutic strategies, their typical indications, advantages, and limitations, providing a practical framework for decision-making in diverse clinical settings. 

**Table 2:** Practical comparison of therapeutic strategies for cornual ectopic pregnancy β-hCG: beta-human chorionic gonadotropin

Strategy	Typical clinical scenario	Main advantages	Main limitations/risks	Evidence profile
Methotrexate (medical management)	Hemodynamically stable, early diagnosis, low β-hCG, small gestational sac, no rupture	Uterine preservation, avoids surgery	Treatment failure, delayed rupture risk, prolonged follow-up	Case reports, small series, reviews
Laparoscopic management (cornuostomy / wedge resection)	Stable patient, surgical expertise available, desire for fertility preservation	Faster recovery, lower morbidity, less blood loss	Technically demanding, requires an experienced team and resources	Retrospective series, technical reports, and limited comparative data
Laparotomy (open surgery)	Hemodynamic instability, rupture, massive haemorrhage, and limited laparoscopic resources	Rapid haemorrhage control, universally feasible	Higher morbidity, longer recovery, potential fertility impact	Comparative studies, historical standard

Laparoscopic surgical techniques

The introduction of laparoscopy has enabled the development and refinement of various surgical techniques for the conservative management and radical treatment of cornual ectopic pregnancy. These techniques aim to reduce surgical morbidity and preserve uterine integrity where clinically feasible. Among these conservative techniques, laparoscopic cornuostomy is one of the most widely used options. It involves making a controlled incision in the cornual myometrium to remove gestational tissue. This is followed by reconstructing the uterine defect using sutures [14, 18, 35].

Evidence supporting laparoscopic cornuostomy mainly derives from retrospective case series and technical reports, with only limited comparative data available. Small series have demonstrated their feasibility and effectiveness in haemodynamically stable patients with smaller pregnancies and no evidence of rupture, suggesting adequate preservation of reproductive potential when performed by experienced teams [14, 36, 37]. In this context, laparoscopic suturing for myometrial reconstruction is considered a key component of the procedure, ensuring haemostasis and potentially reducing the risk of uterine rupture in subsequent pregnancies [14, 36, 37].

Conversely, wedge resection of the cornua or laparoscopic cornuectomy involves the complete removal of the affected segment of the cornua. This technique is typically reserved for cases involving greater structural compromise, larger gestational sacs, or significant bleeding. Evidence for this approach includes case reports, small retrospective series, and technical descriptions from experienced centres, which suggest feasibility even in advanced pregnancies when adequate haemostatic control and safe uterine reconstruction are achieved [10, 16, 38].

The choice of surgical technique depends on various clinical and anatomical factors, such as the size of the gestational sac, the depth of myometrial invasion, the integrity of the uterine horn, the presence of haemoperitoneum, and the surgical team's experience. Despite the growing number of publications, most available data come from observational studies and descriptive series, and there is currently no high-quality comparative evidence establishing the superiority of one technique over another. Therefore, decisions must be individualised on a case-by-case basis [10, 17, 39].

One of the main technical challenges during laparoscopic surgery for cornual ectopic pregnancy is controlling intraoperative bleeding, given the extensive vascularisation of the interstitial myometrium. Various complementary strategies have been described to minimise blood loss, including the intramyometrial injection of diluted vasopressin prior to incision, the use of tobacco pouches or figure-of-eight sutures, and the careful resection of tissue with cold instruments instead of extensive electrosurgery [35, 36, 39].

Advanced technical variants have also been reported, including the use of endoloops, circumferential sutures, and laparoscopic approaches assisted by hysteroscopy or single-port techniques. These approaches are predominantly documented in technical reports and isolated case series, reflecting innovations developed by expert surgeons in specialised centres [37]. These modifications aim to reduce morbidity further, optimise haemostatic control, and preserve as much functional uterine tissue as possible.

Overall, the available evidence suggests that both conservative and radical laparoscopic techniques are safe and effective options for managing cornual ectopic pregnancies in selected patients. However, the literature consistently emphasises that these procedures should only be performed by experienced advanced gynaecological surgeons, given the anatomical complexity of the cornual region and the potential risk of massive haemorrhage [14, 36, 39].

Haemostatic strategies in laparoscopic management

Controlling intraoperative bleeding is one of the main challenges in the laparoscopic management of cornual ectopic pregnancy due to the cornual myometrium's rich and complex vascularisation and its proximity to the uterine and tubal branches. This anatomical feature explains the high risk of massive haemorrhage, even in cases that have not ruptured, making effective haemostasis critical to the success of the minimally invasive approach [10, 40].

One of the most widely described strategies is the intramyometrial injection of diluted vasopressin, which induces transient local vasoconstriction and significantly reduces blood loss during cornuostomy or cornual resection. Multiple studies and case reports have demonstrated that this technique improves visualisation of the surgical field, facilitates controlled dissection of gestational tissue, and reduces the requirement for conversion to laparotomy [19, 41, 42].

In addition, preventive haemostatic suturing techniques have been developed, most notably the purse-string circumferential suture, which is applied at the base of the cornual bulge prior to the incision. This strategy has proven particularly effective in containing blood flow during the removal of the gestational sac, allowing for safer resection, even in larger interstitial pregnancies or those involving significant anatomical distortion [19, 43, 44].

Other authors have described the use of temporary vascular clamping, including applying bulldog clamps to the uterine vessels or temporarily ligating the utero-ovarian ligament, to reduce regional blood flow during critical moments of the procedure. Although technically demanding, these manoeuvres have proven useful in advanced cases or those with a high risk of haemorrhage, provided they are performed by surgeons with experience in advanced gynaecological laparoscopy [45, 46].

Similarly, multi-plane myometrial suturing, performed immediately after resection, is essential not only for definitive haemostatic control, but also for anatomical restoration of the uterine horn and reduction of the risk of uterine dehiscence or rupture in subsequent pregnancies. This careful reconstruction has been consistently associated with good surgical and reproductive outcomes [41, 45, 47].

Finally, the use of advanced surgical energy devices, such as harmonic scalpels or vascular sealing instruments, has been described as an additional tool for optimising haemostasis during incision and cornual resection. However, their use must be balanced with the preservation of myometrial tissue and uterine integrity [27, 46, 45].

Overall, the available literature emphasises that combining pharmacological vasoconstriction, preventive haemostatic sutures, temporary vascular control, and meticulous myometrial reconstruction strategically enables the safe and reproducible laparoscopic management of cornual ectopic pregnancy, even in complex cases, thus establishing this approach as an effective alternative to open surgery [40, 41, 43].

Surgical outcomes and complications

The available clinical series agree that the laparoscopic approach to cornual ectopic pregnancy is associated with favourable surgical outcomes when performed on carefully selected patients by teams experienced in minimally invasive gynaecological surgery. The most consistently reported benefits include low intraoperative blood loss, acceptable surgical times, and short hospital stays, resulting in accelerated postoperative recovery [15, 16, 48].

Conversion rates to laparotomy are generally low in contemporary studies, especially when adequate haemostatic strategies and careful surgical planning are employed. Even in cases involving hemoperitoneum or advanced interstitial pregnancies, several authors have reported the successful completion of the procedure laparoscopically, without the need for an open approach [15, 48, 49].

Intraoperative and postoperative complications are relatively uncommon but may include persistent bleeding, the need for a blood transfusion, persistence of trophoblastic tissue requiring adjuvant methotrexate treatment, and, in exceptional cases, reoperation. However, most of these complications occur in cases of previous rupture or larger pregnancies [18, 40, 50].

Several studies have also evaluated objective safety parameters, such as postoperative haemoglobin decrease, transfusion requirements, and length of hospital stay. The results are comparable to or superior to those historically reported with laparotomy. In this regard, the laparoscopic approach has clearly demonstrated advantages in terms of postoperative morbidity and functional recovery [15, 17, 51].

Overall, the available evidence suggests that laparoscopic management of cornual ectopic pregnancy provides safe and consistent surgical outcomes, with a low incidence of significant complications and a favourable safety profile when used in appropriate cases. These findings have helped to establish laparoscopy as a valid, and even preferred, alternative to laparotomy in centres with adequate experience [15, 16, 49, 51].

Fertility and reproductive outcomes

Preserving fertility is one of the main objectives of surgical management of cornual ectopic pregnancy, particularly in women of reproductive age. The available evidence suggests that the laparoscopic approach, especially when conservative techniques and adequate myometrial reconstruction are used, is associated with satisfactory rates of subsequent pregnancies, comparable to those observed after treatment of other forms of ectopic pregnancy [14,40,52].

Several retrospective series have documented the occurrence of viable and full-term intrauterine pregnancies after cornual resection or laparoscopic cornuostomy. In particular, medium- and long-term follow-up studies have not identified a significant reduction in overall fertility rates when comparing patients treated for cornual ectopic pregnancy with those undergoing salpingectomy for non-interstitial tubal ectopic pregnancy [52,53].

However, the impact of the procedure on subsequent obstetric outcomes has attracted particular attention. The risk of uterine rupture during subsequent pregnancies has been identified, particularly in cases involving extensive cornual resection, significant myometrial scarring, or a short timeframe between surgery and conception. Although rare, this risk has led to recommendations for close obstetric follow-up and individualised pregnancy planning, including consideration of an elective caesarean section for certain patients [16, 40, 52].

Despite the higher rate of elective caesarean sections reported in women with a history of cornual resection, cohort studies have shown that there has been no significant increase in major obstetric complications such as abnormal placentation, preterm delivery, or adverse perinatal outcomes compared to appropriately selected control groups [52, 53].

Likewise, recent research has explored factors that may influence subsequent reproductive outcomes, such as a history of ipsilateral tubal surgery, the intergestational interval, and the surgical technique used. In this context, it has been observed that a short interval between surgery and a new pregnancy may be associated with an increased risk of complications, emphasising the importance of personalised reproductive counselling following the treatment of cornual ectopic pregnancies [54, 55].

Overall, the available evidence suggests that laparoscopic management of cornual ectopic pregnancy can preserve reproductive potential in many patients, leading to favourable obstetric outcomes when cases are appropriately selected and careful obstetric follow-up is provided. However, the rarity of this condition and the heterogeneity of the studies highlight the need for further research using prospective designs and larger sample sizes to more accurately define long-term reproductive risks [17, 56, 57].

## Conclusions

Cornual/interstitial ectopic pregnancy remains a rare but potentially lethal obstetric emergency owing to its diagnostic complexity and high risk of uterine rupture with catastrophic haemorrhage. Early recognition and appropriate management are essential to reduce maternal morbidity and mortality. While the available evidence suggests that laparoscopic management, when performed in carefully selected patients by experienced teams and in adequately equipped centres, is associated with favourable surgical outcomes and reduced postoperative morbidity, this approach should not be interpreted as a universal standard of care. The evidence supporting laparoscopy is predominantly derived from case reports, retrospective series, and specialised tertiary-centre experiences, which may limit its generalisability to broader healthcare settings. Clinical decision-making must therefore be individualised and strongly guided by patient stability, anatomical findings, reproductive wishes, and, critically, the resources and expertise available at the treating institution. In many healthcare systems worldwide, immediate access to advanced minimally invasive surgery, interventional radiology, or specialised blood bank support may not be available; in such contexts, timely laparotomy remains a life-saving and appropriate therapeutic strategy. The primary principle in emergency management must remain maternal safety, with preservation of fertility considered a secondary objective when compatible with clinical stability. Furthermore, management decisions may involve complex ethical considerations, particularly in situations where extensive uterine damage or recurrent risk may render future pregnancy unsafe. In selected cases, definitive surgical management may represent an ethically justified option after appropriate clinical evaluation and counselling.

A practical approach to cornual/interstitial pregnancy, therefore, requires integration of diagnostic accuracy, haemodynamic assessment, resource availability, and surgical expertise rather than reliance on any single technique. Narrative reviews on this topic should aim to support real-world clinical decision-making by acknowledging the diversity of global healthcare contexts and the hierarchy of priorities in obstetric emergencies. In reproductive terms, current data suggest that fertility can be preserved in a substantial proportion of patients following conservative or minimally invasive management, although a low but clinically relevant risk of complications in subsequent pregnancies remains and warrants specialised follow-up and individualised obstetric planning. Given the heterogeneity of the available literature and the predominance of low-level evidence, further prospective studies are needed to refine diagnostic criteria, improve treatment algorithms, define clearer indications for different surgical approaches, and better characterise long-term maternal and reproductive outcomes.
